# Why social justice matters: a context for suicide prevention efforts

**DOI:** 10.1186/s12939-020-01173-9

**Published:** 2020-05-25

**Authors:** Shirley Hochhauser, Satya Rao, Elizabeth England-Kennedy, Sharmistha Roy

**Affiliations:** 1 College of Health and Social Services Building, 1335 International Mall, Suite 326, P.O. Box 30001 MSC 3HLS, Las Cruces, NM 88003-8001 USA; 2 Department of Health & Physical Education, Rhode Island College, 138 Murray Center, 600 Mt. Pleasant Ave, Providence, RI 02908 USA

**Keywords:** Suicide prevention, Social justice, Suicidality, Advocacy, United States, New Mexico

## Abstract

Suicide is among the 10 leading causes of death in the US and has the potential to suddenly change many lives. It often occurs when people are disproportionately affected by societal conditions, including inequities, discrimination, oppression, and historical trauma. We posit that a social justice framework can improve suicide prevention efforts when incorporated into existing strategies because it mandates that inequities be addressed. It does so through education, engagement, advocacy, and action, and can be especially effective in states and nations with high suicide rates and entrenched societal inequities.

In 2016, nearly 45,000 suicides occurred in the US [45]. For each of these, an estimated 30 others attempted suicide [47]. Suicide is the tenth leading cause of death in the US and is one of only three causes that continues to rise [45]. Beyond the number of direct casualties, suicide has a tremendous negative impact on communities and the national economy [47]. These include emotional suffering of families and friends, and economic costs associated with medical care, funeral expenses, and lost productivity [47]. Because of the myriad consequences, suicide is a national concern that must be addressed with urgency and understanding. This is especially true for New Mexico, whose suicide rate has consistently been 50% higher than the US national average and was fourth highest in the country in 2015 [32].

Here, we explore a suicide prevention framework based in social justice. The integration of a social justice context to supplement current suicide prevention efforts in the US can help efforts more fully address suicide-related factors as identified by the socioeecological model (SEM) currently used by the US Surgeon General. A social justice perspective has the potential to improve suicide prevention efforts by reducing stigma, improving access to appropriate healthcare and other services, and creating a supportive, less judgmental, and more inclusive community.

## The Socioecological model

Official suicide prevention efforts in the US began in the late 1950s and have since grown to include various national organizations and task forces, including the National Strategy for Suicide Prevention Task Force, which led to the creation of the 2001 National Strategy for Suicide Prevention, updated by the US Surgeon General in conjunction with the National Action Alliance for Suicide Prevention [47]. Despite these endeavors, suicide rates have remained mostly unaffected for decades and are on the rise in some regions of the country and specific population groups [45]. For example, from 1999 to 2016, suicide rates in the US had increased by almost 30% in the western region and had increased among American Indians and Alaskan Natives [45].

Suicide is often tied to mental health conditions [47]. As a result, suicidology, the study of suicidal behavior and prevention, has been dominated by a clinical-psychiatric approach [4]. However, in 2015, 54 % of suicide victims in 27 states, including New Mexico, did not have a known mental health condition [45]. In fact, mental health status is only one of many factors associated with suicide [47]. Other factors are classified according to the socioecological model outlined in the 2012 National Strategy for Suicide Prevention.

## The four levels of influence

Suicide is an outcome of a complex interaction of multiple components ranging from individual characteristics to environmental influences [47]. These can be found at the four levels established in the socioecological model used by the US Surgeon General: individual, relationship, community, and societal [47]. The individual level of influence includes genetic predispositions, personality traits, and personal experiences, attitudes, perceptions, and views [47]. The relationship level includes family history and interpersonal relationships [47]. The community level takes into consideration interactions with neighborhoods, educational institutions, workplaces, and other community environments, including the healthcare system [47]. The societal level considers media influences and the impact of governmental policies and decisions, including policies related to access to lethal means [47]. Within each of these are embedded factors that can either increase or reduce a person’s susceptibility to suicide, known as risk and protective factors [47].

## Risk and protective factors

Risk factors are characteristics that increase an individual’s potential to develop suicidal ideation and engage in suicidal behaviors [30]. These can be short-term crises or long-term sources of stress [30]. They exist within all four levels of influence and can include substance abuse, family history of suicide, barriers to healthcare, and problematic media portrayals of suicide [47]. Protective factors are characteristics that make development of suicidal ideation and behaviors less likely [30, 38]. They provide sources of support and promote strong connections between individuals and the world around them [30].

Protective factors also exist at every level and include healthy coping skills, supportive social interactions, safe community environments, and affordable healthcare [38, 47]. According to the 2012 National Strategy for Suicide Prevention, suicide is less likely to occur when risk factors are minimized and protective factors are maximized, Suicide prevention strategies need to incorporate multiple perspectives to address as many of these factors as possible [47].

Although suicide can affect all, certain groups have been impacted more than others. These include American Indians and Alaska Natives (AIAN), members of the military, including veterans, men in their midlife, and older white men, as well as LGBTQ individuals, people who have lost loved ones to suicide, are in justice and/or child welfare systems, engage in non-suicidal self-injury, attempted suicide in the past, have chronic medical conditions, are in grief or enduring severe pain, and/or have mental and/or substance abuse disorders [47]. Each of these groups has a wide range of specific risk factors; however, here we focus on factors that are impacted by unjust socioeconomic conditions, negative attitudes, and stigmas concerning suicide. Many are interrelated and can be experienced concurrently, which can make the ability to combat suicidality on an individual level more difficult and even impossible.

Risk factors experienced by AIAN individuals, as well as those in other ethnic and minority communities, include discrimination, limited culturally-appropriate mental health service access, and historical trauma [47]. Discrimination can include individual- and institutional-level discrimination. Institutional discrimination can come in the form of laws and public policies that create inequalities or omissions from benefits or protections [33]. This type of discrimination can lead to inadequate and inappropriate care of vulnerable populations [33]. For example, widely-used preventive interventions and currently-identified suicide-related risk (and protective) factors may not necessarily be applicable to people of color, because they were tested in White-majority communities [52]. Institutional discrimination can therefore be a reason why AIAN and other minorities have limited access to culturally-appropriate and compassionate mental and physical health services that could help them tackle issues like historical trauma, a known threat to physical and mental health [38]. Individuals with mental and/or substance abuse disorders, people in justice and/or child welfare settings, and LGBTQ persons can also suffer from a lack of appropriate mental health services due to institutional discrimination [33, 47].

Members of sex and gender minorities are more likely to encounter healthcare providers who either do not have the proper training to address specialized health needs or have personal prejudices and biases that interfere with their ability to deliver timely and non-judgmental care [33]. LGBTQ individuals are also at risk for suicide due to minority stress, which stems from cultural and social prejudices they face daily because of their minority sexual orientation and gender identity status [47]. This also contributes to their susceptibility to suicide contagion, due to media coverage that presents suicidal behavior as a normative reaction to minority stress [47].

Social disconnection and reluctance to seek help are risk factors experienced by men in their midlife, who account for the majority of suicides in the US, and older white men, whose suicide rate is almost three times higher than the rate of the general population [47]. Although white men are not usually thought to suffer consequences of social injustice, studies have proposed that social pressures associated with maintaining one’s reputation and conforming to cultural ideals of masculinity play a part in increased risk of suicide for this demographic [10].

Unequal economic conditions can also exacerbate risk factors and limit development of protective factors, especially for people who experience many of the above-mentioned conditions concurrently. For example, poverty has both short- and long-term effects on health and sense of security because income is necessary for acquiring and maintaining resources like food, shelter, and healthcare [35]. People with serious and acute emotional and psychological distress are less likely to have health insurance because of an inability to afford and/or acquire insurance [8]. Even people with insurance often find they do not have adequate coverage for psychiatric treatment and/or therapeutic services, either because they cannot find professionals who accept their insurance, or because their insurance has high copayments and deductibles [8]. Finally, inability to provide for family or themselves can also lead to feelings of hopelessness and negativity, particularly for men [35].

## A different perspective

A clinical-psychiatric framework works well in addressing factors within the individual level of influence. Therapeutic interventions based on this framework include psychotherapy and medication, which address risk factors like mental health disorders, substance abuse, and aggression, while also promoting protective factors like healthy coping skills and open communication [31]. However, limitations exist because counselors, therapists, and psychiatrists can only help at-risk individuals if these individuals are able to afford and consistently access adequate physical and mental health care [44]. Additionally, current mainstream studies and practices of suicide prevention tend to privatize pain and individualize suicide by focusing on risk factors like short-term crises, family history of suicide, mental health disorders, and substance abuse. This can propagate stigma and discrimination and therefore make it less likely for at-risk individuals to seek help, even if they can afford it [39]. Furthermore, because current approaches to suicide prevention are often based on psychiatric and individual dynamics, they do not adequately acknowledge or address other risk factors discussed previously [28]. Although therapeutic interventions can help individuals deal with how they feel about their unjust circumstances, a clinical-psychiatric approach alone cannot adequately address social injustice.

Nations, including the US, have an implicit obligation to improve environmental conditions that correlate with higher rates of suicide because one of the primary goals of a political state is to protect its citizens from violence and death [4]. A social justice framework brings this obligation to the forefront by making community members aware of the societal status quo, the harmful effects of social injustice on at-risk individuals, their own direct or indirect role in maintaining these dangerous inequities, and their ability to change so their fellow citizens can have equal opportunity to dignified lives [4]. By encouraging a critical analysis of the social, economic, relational, and cultural contexts within which suicide occurs, a social justice framework can minimize risk factors and increase protective factors at the community and societal levels of influence, thereby allowing suicide prevention efforts to be more comprehensive, effective, and long-lasting.

## A social justice framework

A social justice framework, in general, is composed of three main concepts: (1) acknowledgment of inequality and oppression, (2) assumption of involvement, and (3) obligatory addressal of the issues through responsible action [43]. People seeking to implement a social justice framework must first acknowledge that there is an inequitable distribution of power, resources, and access within society [43]. Beyond acknowledging that social injustice exists, people must also recognize that everyone and everything within the system participates in maintaining the societal status quo, however unintentionally [43]. Finally, action should be taken to mitigate negative effects of social inequality and promote social, political, and economic parity [43]. Though a social justice framework can be applied to multiple social problems, it is particularly suited to and important for suicide prevention efforts because of its multifaceted nature. It is also based on the recognition that inequality and oppression contribute to the prevalence of suicide and on the understanding that we as individuals and communities have the power to advocate for and create sustainable change that addresses suicide in thoughtful and compassionate ways.

If we follow the general social justice framework presented by Smith et al. [43], the first aspect of a social justice-based framework for suicide prevention should be the acknowledgment of the insidious effect of social injustice on suicidal behavior [43]. Socioeconomic inequality cultivates social injustices, including poverty, homelessness, racism, sexism, homophobia, ageism, refugee status, and historical trauma that inordinately affect minority and marginalized groups and communities and contribute to suicidality, especially in already-vulnerable people [11, 13–15, 18, 22, 29, 51, 53]. In some cultural settings, such as Maori culture, the power of current sociocultural forces disrupts traditional understandings and social modelings and increases the risk of suicidality [16].

Despite these socioeconomic risk factors, suicide is primarily viewed as a moral failure of individuals, e.g., as a sign of “weakness,” “cowardice,” “badness,” “mental illness,” or “admission of failure” [12, 24, 40], This perspective has become normalized in many societies, and reflects discomfort with and avoidance or rejection of the topic [24, 40]. This view of suicide has been documented at all socioecological levels, including individuals who self-stigmatize [24, 40, 41]; families, peers, and other social groups [9, 24]; community [1]; policy (e.g., insurance companies, policies, and personal attitudes affecting coroners’ official declarations of cause of death; Noble, 2010 [46];); and media messages, including mass and social media [21, 25, 26].

This perspective is structurally reinforced. For example, insurance companies may legally deny coverage for death resulting from suicide [34]. Commonly-offered suicide prevention and intervention trainings such as Applied Suicide Intervention Skills Training (ASIST) typically focus on individuals rather than systemic or contextual factors (e.g., Evans & Price, 2012 [17]; Shannonhouse, et al., 2017 [42];). In some cases, institutionalized discomfort and/or lack of familiarity with the topic of suicide has led to avoidance of the topic by school-based counselors in conversations with students, potentially due to assumptions made in professional education curricula [3, 23].

Because suicide is still largely seen as an entirely personal matter, everyone - from healthcare providers to politicians to educators - must become more aware of how suicide can be a response to external conditions that are outside of an individual’s immediate control. We need public health strategies based on a social justice framework that allow for a deeper and non-judgmental understanding of the underlying reasons for suicide prevalence in specific communities and certain regions of the country.

A contemporary way of raising consciousness is through​ use of popular cultural media. As previously mentioned, media can serve as a major factor that can increase the risk of contagion through sensationalization, romanticization, and/or normalization of suicide, especially when reporting on suicide completion by celebrities [5, 21, 26, 53, 54]. However, it can also be a powerful tool that provides information on how and where to seek help, and highlights ways to cope with life stressors and what can be done collectively and intersectionally to improve suicide prevention efforts. This is especially true when suicide-reporting guidelines are closely followed, suicide literacy is promoted, and/or limitations on sales of suicide-related products are legally instituted to support prevention efforts [5, 19, 50, 54]. If not, imitative suicide rates can be high enough to alter long-standing epidemiological patterns [2, 6].

Although media can be helpful in dispensing basic information and context to the general public, more specific tools should also be utilized. For example, in order to more effectively raise consciousness and understanding of suicide, education tools should also be culturally appropriate when possible. This can include memoirs and *fotonovelas*, booklets with posed photographs and easy-to-understand text bubbles that have been used to provide cost-effective health education to Hispanic populations and underserved populations with limited health literacy [20, 37]. These have been used to improve knowledge about depression and have been proposed as a channel to help combat mental health stigma on college campuses [37]. Since *fotonovelas* can be adopted to any culture, and have already been used to address suicide risk factors like mental health disorders, they are an ideal channel to disseminate information and explain how social injustice may contribute to suicide prevalence [37]. By raising awareness and educating people about how social injustice can worsen suicidal ideation, attempts, and completions, at-risk individuals can be helped to realize that they are not alone and that they are not at fault.

The second concept is recognition of personal and collective involvement in the propagation of socially unjust conditions. This can be achieved by promoting community engagement, social activism, and advocacy because they will help people realize they have the capacity and power to combat and change social injustice and pervasive outcomes such as suicide. People who benefit from current societal attitudes, economic conditions, and public policies often fail to see how they are protected by society, just as they fail to realize how their neighbors and friends are harmed by it [35]. Therefore, it is important to actively mobilize bystanders and to connect people and communities so the unaffected can learn and help contribute to humanistic change [44].

The third and final concept of the social justice framework presented by Smith and colleagues is action. Action should be taken to mitigate negative effects of injustice and inequality; promote social, political, and economic equity and personal agency; and promote a sense of collective and communal responsibility [43]. We recommend a broadened scope of research to explore the implementation of a social justice framework, evaluate its effectiveness. and provide directions for suicide prevention program designers and policy-makers. College campuses are well-suited for this investigation because of the diversity of their student populations, which include students with disabilities, ethnic and/or sexual minority students, and those who are of undocumented immigrant status [37]. Finally political action must be taken to ensure implementation of policy and legislative changes that actively reduce resource gaps and highlight the humanity of those experiencing suicidality [36].

Currently, New Mexico has one of the highest suicide rates in the country [32]. The state is predominantly rural, culturally diverse, tribal, and economically disadvantaged, and shares a border with Mexico. Existing socioeconomic hardships, limited access to healthcare resources, discrimination, oppression, and disconnectedness all contribute to the high rate of suicide [49]. For these reasons, states such as New Mexico can be ideal for implementing a social justice framework.

A complex interaction of political, social, and economic disparities contribute to and maintain unjust conditions. In New Mexico, these disparities include lack of firearm education and regulations, and stigmas surrounding people who are poor, homeless, and unemployed [35]. The combination of these disparities, conditions, and circumstances may be a critical reason why New Mexico has experienced increased suicide rates, which makes the implementation of a social justice framework essential. This framework has potential to intentionally raise awareness and educate people and communities to become aware of underlying and upstream contributors to suicide, to engage in activism and advocacy, to become empowered change agents, and to push for micro/individual-level changes and/or policy and legislative changes that are rooted in human dignity and compassion for those who are feeling the burden of suicide.

The efforts of an Athabaskan tribe in New Mexico are an example of how a social justice framework that integrates greater understanding of socioeconomic conditions can be effective. Community engagement that addresses socioeconomic problems within the tribe enhances collaborations between the tribal council, tribal leaders, citizens, and institutions such as Indian Health Service (IHS), which in turn increases collective and individual awareness of suicidal behaviors and efficacy. The program was initiated through collaboration by the tribal council, community, and IHS in 1990 following an increase in suicides by tribal adolescents and young adults [28]. The tribe at the center of the study was located in a rural, isolated, impoverished area. Eighty percent of the tribe who were aged sixteen and older were either wholly unemployed or had limited seasonal employment; most were unemployed. The program’s goal was to reduce the incidence of adolescent and young adult suicidal behavior through community education and awareness about suicides and related behavioral issues [28].

The program included multiple community-based interactive workshop sessions that examined questions about problems and issues facing the community, barriers to resolving these issues, and possible solutions. Problems included alcoholism, other substance abuse, domestic violence, child abuse, and unemployment. Members of the community were educated on risk factors for suicide and high-risk individuals were linked to services and community-based prevention-focused activities, using a systems-based approach. Workshop sessions were followed by screening and clinical interventions, community events, social services, and school-based prevention programsthat involved stakeholders such as tribal leaders and elders, family members, and health professionals. Additional program components included local surveillance and outreach, diligent record-keeping, consistent evaluation, community-based education, integration of appropriate violence and substance abuse prevention services, a team-based approach, a behavioral health base, cultural relevance, and integration into the tribal infrastructure. Community members were involved at all levels of the program, from planning through implementation. All community members were involved in the program in some capacity, whether as part of peer training, as informal counselors, or as program advocates. During the planning process, community members indicated that suicidality could not be addressed as a problem of individuals, as multiple social risk factors contributed to the problem [28].

According to the outcome evaluation, the total number of self-destructive acts declined within the targeted age group by 73% across the program’s span. The clearest drop in frequency was of suicidal gestures, although suicide attempts were also significantly lowered [28]. By acknowledging and addressing socioeconomic conditions that contributed to tribal youths’ suicidal behaviors, the program fostered active community involvement, which in turn promoted the de-individualization and de-stigmatization of suicide. The evaluation suggested that suicide prevention programs need to identify and mitigate underlying social, psychological, and developmental issues. The authors also highlighted the importance of active and ongoing community involvement and participation in the development and implementation of such programs. These recommendations reflect the principles of a social justice framework: Acknowledgment of the insidious effect of social injustice on suicidal behavior, the relevance of involvement, and use of thoughtful, community-driven actions to address issues. This program can therefore serve as a template to craft social-justice-based approaches to suicide prevention, especially because of its promising results.

Other suicide prevention projects have also indicated a need to address historic and multiple social factors in suicide prevention. Walls, Hautala, and Hurley [48] found that Aboriginal First Nation community members in multiple sites identified historical trauma and normalization of suicide as key risk factors. Loss of a sense of tribal identity and communication barriers leading to social disconnection were identified as underlying causes of this normalization, and a need to return to pre-colonial forms and levels of connectedness was highlighted as a means of lowering suicide risk.

## Conclusions

Unjust and discriminatory societal conditions and attitudes often contribute to suicides. Some groups and/or communities are disproportionately affected by these societal conditions, particularly those that have experienced historical trauma, such as tribal and other marginalized communities. Generally, current suicide prevention efforts do not incorporate ways to adequately acknowledge or address these disparities or discriminatory practices. Therefore, we posit that a social justice practice framework is needed, especially in states like New Mexico and in tribal and aboriginal communities, where at-risk individuals face a host of interrelated socioeconomic and other issues that increase vulnerability to suicide.

By highlighting injustices, this framework pushes people to question and examine the historical, socioeconomic, cultural, and other factors that contribute to suicide, which can in turn help promote protective factors and reduce risk factors identified by the socioecological model currently utilized by the United States Surgeon General [47] and the WHO (2020) in its Global Campaign for Violence Prevention. A social justice framework not only acknowledges that a variety of external social and environmental influences can detrimentally affect individual suicidality, but also proposes that these exogenous factors can be altered [4]. In doing so, this framework promotes community education and engagement, which in turn helps combat the privatization of pain and the individualism of suicide [39]. It also has the capacity to reduce stigmatization of and negative judgments towards suicide, which can then create spaces for disclosure, dialogue, and help-seeking behaviors [44]. Although not all instances of suicide are responses to social injustice, incorporating this framework into suicide prevention strategies and programs can foster a more nuanced understanding of the problem and enhance the effectiveness of prevention efforts [39].

Although a social justice framework has the theoretical and intuitive potential to improve suicide prevention efforts, rigorous evaluations are necessary to document its efficacy. Previous systematic reviews of suicide prevention strategies and interventions stress the need for increases in the number and quality of evaluations to optimize the use of limited resources [7, 27]. Currently, only education of physicians in identifying at-risk individuals and restricting access to lethal methods has been shown to effectively reduce suicide rates [27]. Other social justice-based interventions, like stigma reduction campaigns, public education efforts, awareness campaigns, inclusion/connectedness strategies, and others need further testing to evaluate their efficacy [27].

## Data Availability

Not applicable.
